# Chromosomal instability induced by CRISPR/Cas9: implications for pancreatic cancer therapy

**DOI:** 10.1172/JCI206028

**Published:** 2026-05-15

**Authors:** Li-Chan Chang, Christine E. Eyler, Chang-Lung Lee

**Affiliations:** 1 Department of Radiation Oncology and; 2 Department of Pathology, Duke University School of Medicine, Durham, North Carolina, USA.

## Abstract

Clinical management of pancreatic cancer (PC) remains severely limited, primarily due to the complex tumor microenvironment. Emerging DNA damage–targeted strategies have demonstrated considerable therapeutic potential in PC. In this issue of the *JCI*, Teh et al. employed cancer-specific multitarget sgRNAs to induce DNA double-strand breaks (DSBs), resulting in lethal effects in PC cells. Integrative bioinformatic and cytogenetic analyses revealed that CRISPR/Cas9-mediated DSBs provoked persistent chromosomal instability, ultimately leading to chromosome catastrophe and cell death. Compared with equivalent radiation-induced DSBs, these sgRNAs exhibited superior cytotoxicity and were able to eliminate cells resistant to a specific sgRNA via subsequent targeting at distinct genomic sites, highlighting a promising and innovative precision therapeutic approach for clinical treatment of PC.

## Clinical challenges in pancreatic cancer therapy

Pancreatic cancer (PC) has the highest mortality-to-incidence ratio among major malignancies (1), largely due to late diagnosis, intrinsic cellular/genetic factors, and a dense, hypovascular, immunosuppressive tumor microenvironment that limits drug penetration (2). Localized, nonmetastatic disease may be treated by a combination of aggressive chemotherapy and surgery and/or radiotherapy (RT). However, due to the anatomic features of this tumor type, not every patient with nonmetastatic, localized disease is a candidate for definitive surgery. Other local therapies, like ablative-dose radiation, are also limited by the proximity of these tumors to sensitive structures, so even localized PC is likely to progress to metastatic fatal disease despite aggressive definitive treatment. Current FDA-approved first-line chemotherapies for metastatic disease provide modest survival benefits but are hampered by systemic toxicity and resistance (3–5). There is a clear need for innovative strategies to overcome these therapeutic limitations.

## Therapeutic strategies based on DNA damage response

**Figure 1:**
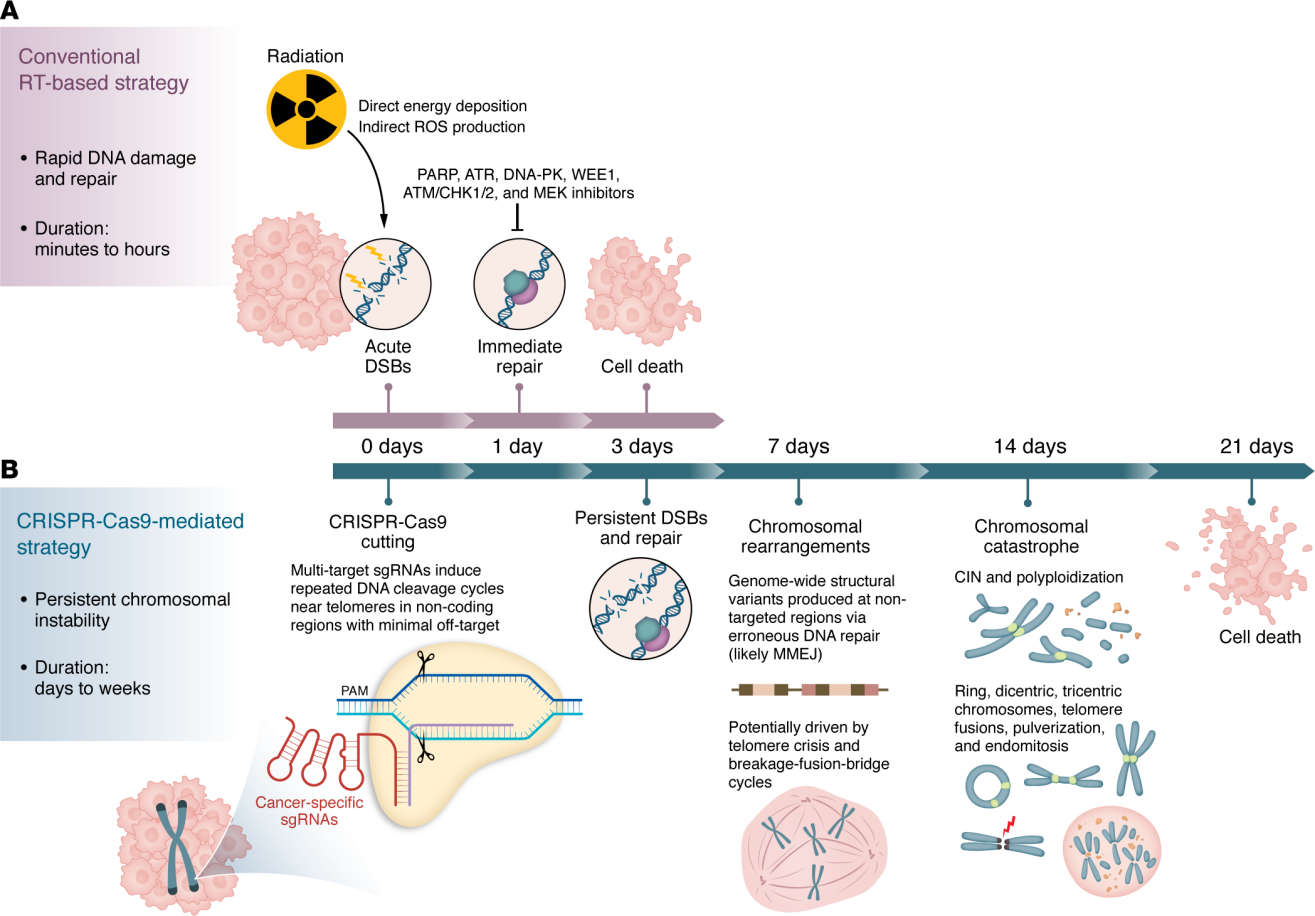
Anticancer strategies based on DNA DSBs induced by RT and CRISPR/Cas9 systems. (**A**) Conventional RT induces DNA damage in cancer cells through both direct ionization and indirect effects mediated by ROS, among which DSBs are the most lethal form. This process occurs rapidly (within hours), and DNA repair pathways — including nonhomologous end joining, homologous recombination, and microhomology-mediated end joining (MMEJ) — are promptly activated. Insufficient or failed repair subsequently leads to cell death through multiple pathways. Clinically, therapeutic efficacy can be enhanced by increasing the extent of DSBs and/or inhibiting DNA repair mechanisms. (**B**) The strategy reported by Teh et al. (12) induced simultaneous CRISPR/Cas9-mediated DSBs using cancer-specific protospacer adjacent motif–guided (PAM-guided) multitarget sgRNAs delivered into Cas9-expressing cancer cells. This approach induced persistent DSB formation and continuous DNA repair over several days, leading to genome-wide structural variations and ongoing chromosomal rearrangements. As chromosomal instability (CIN) accumulated and reached a critical threshold, cells underwent polyploidization and developed severe chromosomal aberrations — a process collectively referred to as chromosome catastrophe — ultimately resulting in delayed cell death via apoptosis.

Cancer cells are exposed to intrinsic and extrinsic stresses that induce DNA damage, such as replication stress, reactive oxygen species (ROS), DNA repair defects, and hypoxia (6). The balance between DNA damage and repair is critically important. Moderate DNA damage can promote chromosomal instability in cancer cells, leading to gene mutation accumulation and drug resistance. In contrast, excessive DNA damage can trigger mitotic catastrophe, cell cycle arrest, and apoptosis, ultimately killing cancer cells. RT exploits this balance by inducing lethal DNA double-strand breaks (DSBs) to suppress tumor growth (Figure 1A). Conventional fractionated RT for PC delivers 1.8–2 Gy per fraction alongside radiosensitizing chemotherapy to a total of 50–54 Gy. Though higher doses of radiation (e.g., 75–100 Gy, conventionally fractionated) or ultrahypofractionated RT courses (40 Gy or more delivered in 5 fractions) can improve treatment responses, in many cases the maximum deliverable doses are limited by proximity to radiation-sensitive structures like the small bowel/duodenum and stomach (7). For most tumors, these limitations render the safe range of RT doses inadequate to cure localized PC without other treatment modalities (8). Recently, RT technologies like SBRT/SABR, FLASH RT, and MR-guided LINAC have been major focuses of development, but these approaches are still hampered by anatomic maximum dose safety limitations (9–11). In this issue of the *JCI* (12), Teh et al. reported a CRISPR/Cas9-mediated tumor-specific strategy that induces DSBs and widespread chromosomal instability in PC cells, demonstrating greater cytotoxicity than similar numbers of radiation-induced DSBs (Figure 1B).

## Precision cancer suppression by CRISPR/Cas9-induced DSBs

CRISPR/Cas9 is a powerful genome-editing technology that was recognized by the 2020 Nobel Prize. The Cas9 nuclease, guided by a sgRNA, precisely cleaves target DNA sequences to generate DSBs, which are subsequently repaired through nonhomologous end joining or homology-directed repair (13). Although most studies have focused on the downstream genomic editing outcomes of DNA repair, relatively few have explored the direct cytotoxic potential of CRISPR-induced DSBs.

Building on their previous work on somatic mutation–derived protospacer adjacent motifs for CRISPR/Cas9-selective targeting in PC (14), Teh and colleagues leveraged genomic sequence redundancy to design a series of multitarget sgRNAs containing 2–16 cutting sites located within noncoding regions of the genome, thereby minimizing disruption of essential genes (12). Given that p53-dependent DNA damage responses may limit CRISPR/Cas9 editing efficiency (15), the team conducted experiments using two PC cell lines harboring inactivating *TP53* mutations. This choice is clinically relevant, as approximately 70% of PC cases exhibit *TP53* inactivation (2). They hypothesized that simultaneous induction of multiple Cas9-mediated DSBs would drive cytotoxicity and chromosomal instability in PC cells.

The results showed that cytotoxicity was observed specifically in Cas9-expressing cells transduced with multitarget sgRNAs. In general, sgRNAs with a higher number of target sites induced stronger growth inhibition, whereas patient-derived fibroblast cell lines exhibited minimal toxicity due to the absence of corresponding target sites. Whole-genome sequencing (WGS) and deep, next-generation sequencing (NGS) further confirmed that most mutations originated from the intended target sites, with minimal off-target effects, suggesting tumor-selective activity. Moreover, subcutaneous injection of these CRISPR/Cas9-treated PC cells into mice resulted in significantly reduced tumor growth and suppressed liver metastasis without affecting body weight, and hydrodynamic injection was used as a proof-of-concept delivery approach.

## Chromosomal instability after CRISPR/Cas9-induced DSBs triggers cell death

Mechanistically, Teh et al. demonstrated that simultaneous multitarget CRISPR/Cas9 cleavage did not immediately induce cell death but instead produced a pronounced delayed cytotoxic effect (12). Combined WGS and cytogenetic analyses revealed a temporal sequence of events: chromosome breaks, chromatid breaks, and radial formations were observed as early as day 1, and DNA scission and repair peaked at days 3–4. These multiple DSBs subsequently triggered genome-wide structural variations beyond the intended target sites, resulting in sustained genomic instability. Polyploidy peaked around day 10, and chromosomal rearrangements reached a maximum by day 14, accompanied by increasingly complex chromosomal abnormalities, including ring chromosomes, dicentric and tricentric chromosomes, telomere-to-telomere fusions, and chromosome pulverization. These events culminated in chromosome catastrophe, initiating apoptosis, with peak cell death observed by day 21.

These phenomena were closely associated with the design of the multitarget sgRNAs, whose target sites are located near telomeres. This positioning may induce telomere crisis (16), causing end-to-end chromosome fusions and breakage-fusion-bridge cycles (17). While chromosomal instability can confer advantages to cancer cells, excessive imbalance ultimately harms them. This highly innovative strategy — wherein accumulating multiple DSBs leads to sustained chromosomal instability that causes structural collapse and ultimately cell death — differs fundamentally from conventional approaches that rely on increasing DNA damage and inhibiting repair to induce cell death.

For patients with PC, current therapeutic strategies targeting DSBs primarily focus on inhibiting DNA repair pathways. These include FDA-approved poly (ADP-ribose) polymerase (PARP) inhibitors for patients with BRCA1/2 mutations, as well as other inhibitors combined with chemotherapy or RT, such as ATR inhibitors, DNA-PK inhibitors, WEE1 inhibitors, ATM/CHK1/2 inhibitors, and MEK inhibitors (3). Therefore, a deeper investigation into DNA repair mechanisms in the context of CRISPR/Cas9-induced DSBs is critically important. Notably, in this study, multitarget sgRNA–transduced cells exhibited a higher proportion of breakpoints with 1–20 bp microhomologies compared with nontargeting controls, indicative of microhomology-mediated end joining involvement in DSB repair. Future analyses of relevant DNA repair regulators, such as POLQ, PARP1, and MRE11, are warranted and may enable combination strategies that pair CRISPR/Cas9-induced DSBs with DNA repair inhibitors to achieve enhanced anticancer effects (18).

## Clinical potential of CRISPR/Cas9-induced DSBs compared with RT

To further evaluate the potential clinical applications of multitarget sgRNAs, Teh et al. compared CRISPR/Cas9-induced DSBs with DNA damage caused by ionizing radiation (12). Their results showed that multitarget sgRNAs with approximately 5 target sites produced clonogenic inhibition comparable to a clinically relevant single 2 Gy fraction of RT, while sgRNAs with 12 target sites achieved cytotoxic effects similar to radiation doses exceeding 4 and 6 Gy. For perspective, 2 Gy of radiation induces approximately 20–40 DSBs per cell, as measured by γ-H2AX foci (19). Interestingly, at comparable levels of cell killing, the number of γ-H2AX foci induced by CRISPR/Cas9 was lower than that caused by irradiation, indicating that comparable cytotoxic effects accompanied fewer sites of damage. The team also demonstrated that this difference could be attributed to the sustained generation of DSBs by CRISPR/Cas9, whereas radiation induces DNA damage in a rapid and transient manner that can be immediately resolved by cellular DNA repair mechanisms.

Nevertheless, the clinical value of RT should not be overlooked. Beyond inducing DSBs, ionizing radiation also generates large amounts of ROS, leading to protein oxidation and lipid peroxidation, which can cause membrane damage, mitochondrial dysfunction, and additional cellular stress. Moreover, RT exerts important immunomodulatory effects, including the release of damage-associated molecular patterns, which can promote antitumor immune responses (20). As discussed later in this Commentary, external beam RT does not suffer from the delivery issues that must be overcome for gene delivery of CRISPR/Cas9 modules to PC. Unlike CRISPR-based approaches, RT does not rely on intracellular delivery, representing a key advantage in clinical implementation.

Therefore, a promising future research direction would be to evaluate the application of CRISPR/Cas9-induced DSB strategies in tumor cells that exhibit resistance to RT or even as a means of radiosensitizing PC cells. Notably, the study demonstrated that PC cells surviving an initial round of multitarget sgRNA treatment could still be further targeted by delivering additional sgRNAs directed at different genomic loci. This approach can induce additional DNA breaks and may reduce the likelihood of stable resistance developing.

## Translational barriers and future perspectives

Despite the promising findings of this study, several limitations and concerns remain. First, even under controlled cell line conditions, the cytotoxic effects varied substantially among different multitarget sgRNAs, likely reflecting differences in target site number, genomic context, and cutting efficiency. Given the substantially greater heterogeneity observed in clinical tumors, validation in more physiologically relevant models, such as patient-derived organoids or xenografts, would further strengthen the translational potential of this approach. In addition, integrating single-cell RNA sequencing and spatial transcriptomics could help delineate heterogeneous cellular responses and identify key regulators underlying differential sensitivity to CRISPR/Cas9-induced DSBs across tumor cell populations (21).

Second, the current in vivo evaluation lacks a fully representative tumor microenvironment. Orthotopic pancreatic injection models should be employed to more accurately assess tumor growth and metastatic progression while considering the influence of the immune system. Furthermore, intracellular CRISPR/Cas9 activity is a critical determinant of tumor suppression, highlighting the importance of developing efficient and precise delivery strategies. Given the dense stromal barrier characteristic of PC, overcoming this obstacle remains a critical challenge.

Implementation of Teh et al.’s CRISPR/Cas9 approach would require efficient delivery of gene-editing components, such as nanoparticles or viral vectors (22). The authors investigated splenic injection as a hepatic delivery strategy to target liver metastases of PC; however, alternative approaches would be required for clinical translation. Although not yet widely adopted, several approaches have been explored in clinical or preclinical settings, including intravenous delivery of liver-tropic gene therapy vectors or nanoparticles (23), hepatic artery infusion for patients with liver-dominant metastases, and direct intratumoral or intraoperative administration of gene therapy vectors to tumor sites or tumor beds (24). Collectively, achieving tumor-specific delivery while minimizing off-target editing in normal tissues will be a critical focus for future development.

Finally, long-term safety remains a major concern for gene editing–based therapies, including risks such as off-target mutations, chromosomal rearrangements, genomic instability, immune responses, and damage to nontumor tissues, which could lead to tumorigenesis or impaired organ function (25). Reliance solely on cell line experiments and sequencing analyses is insufficient to fully assess the potential risks of CRISPR/Cas9-induced DSBs. Comprehensive and longitudinal in vivo biosafety assessments will likely be essential to support future clinical translation.

## Conclusion

Overall, this study pioneers a distinct therapeutic strategy for the clinical treatment of PC. Unlike conventional CRISPR/Cas9 approaches that primarily focus on editing gene expression, this strategy employs cancer-specific multitarget sgRNAs designed to induce multiple DNA cleavage sites, thereby generating widespread structural variations and sustained chromosomal instability. These extensive DNA damages ultimately culminate in chromosomal catastrophe and cell death, exhibiting greater cytotoxicity than irradiation-induced DSBs. Furthermore, the sequential delivery of sgRNAs targeting new cleavage sites can mitigate the emergence of resistant subpopulations, moving closer to the ultimate goal of precision medicine. However, overcoming delivery and safety barriers will be essential before this strategy can be translated into clinical practice.

## Conflict of interest

The authors have declared that no conflict of interest exists.

## Funding support

- CLL by the Whitehead Scholar Award from Duke University School of Medicine.

## Version 1 05/15/2026

Electronic publication

## References

[1] Siegel RL (2026). Cancer statistics, 2026. CA Cancer J Clin.

[2] Kung HC (2025). The tumour microenvironment in pancreatic cancer - new clinical challenges, but more opportunities. Nat Rev Clin Oncol.

[3] Hu ZI, O’Reilly EM (2024). Therapeutic developments in pancreatic cancer. Nat Rev Gastroenterol Hepatol.

[4] Von Hoff DD (2013). Increased survival in pancreatic cancer with nab-paclitaxel plus gemcitabine. N Engl J Med.

[5] Conroy T (2011). FOLFIRINOX versus gemcitabine for metastatic pancreatic cancer. N Engl J Med.

[6] Groelly FJ (2023). Targeting DNA damage response pathways in cancer. Nat Rev Cancer.

[7] Reyngold M (2019). Ablative radiation therapy for locally advanced pancreatic cancer: techniques and results. Radiat Oncol.

[8] Springfeld C (2023). Neoadjuvant therapy for pancreatic cancer. Nat Rev Clin Oncol.

[9] Liu X (2023). Clinical application of MR-Linac in tumor radiotherapy: a systematic review. Radiat Oncol.

[10] Mills BN (2022). Modulation of the human pancreatic ductal adenocarcinoma immune microenvironment by stereotactic body radiotherapy. Clin Cancer Res.

[11] Ma Y (2025). Advancing proton FLASH radiation therapy: innovations, techniques, and clinical potentials. Int J Radiat Oncol Biol Phys.

[12] Teh SSK (2026). Simultaneous CRISPR/Cas9-induced double-strand breaks are lethal in models of pancreatic cancer. J Clin Invest.

[13] Li T (2023). CRISPR/Cas9 therapeutics: progress and prospects. Signal Transduct Target Ther.

[14] Teh SSK (2024). CRISPR-Cas9 for selective targeting of somatic mutations in pancreatic cancers. NAR Cancer.

[15] Haapaniemi E (2018). CRISPR-Cas9 genome editing induces a p53-mediated DNA damage response. Nat Med.

[16] Chakravarti D (2021). Telomeres: history, health, and hallmarks of aging. Cell.

[17] Zhang CZ (2026). A breakage-replication/fusion process explains complex rearrangements and segmental DNA amplification. Nat Genet.

[18] Sfeir A (2024). Microhomology-mediated end-joining chronicles: tracing the evolutionary footprints of genome protection. Annu Rev Cell Dev Biol.

[19] Bonner WM (2008). GammaH2AX and cancer. Nat Rev Cancer.

[20] Guo S (2023). Radiation-induced tumor immune microenvironments and potential targets for combination therapy. Signal Transduct Target Ther.

[21] You Y (2024). Systematic comparison of sequencing-based spatial transcriptomic methods. Nat Methods.

[22] Hii ARK (2024). Advanced strategies for CRISPR/Cas9 delivery and applications in gene editing, therapy, and cancer detection using nanoparticles and nanocarriers. J Mater Chem B.

[23] Outmani L (2025). The landmark series: hepatic arterial infusion pump chemotherapy for colorectal liver metastases and intrahepatic cholangiocarcinoma. Ann Surg Oncol.

[24] Aguilar LK (2015). Gene-mediated cytotoxic immunotherapy as adjuvant to surgery or chemoradiation for pancreatic adenocarcinoma. Cancer Immunol Immunother.

[25] Aussel C (2025). The hidden risks of CRISPR/Cas: structural variations and genome integrity. Nat Commun.

